# Supplementary figures and images for: Forecastability of infectious disease time series: are some seasons and pathogens intrinsically more difficult to forecast?
Source: PLoS Comput Biol. 2026 Apr 15;22(4):e1014175. doi: 10.1371/journal.pcbi.1014175 (PMC13102302; doi:10.1371/journal.pcbi.1014175)

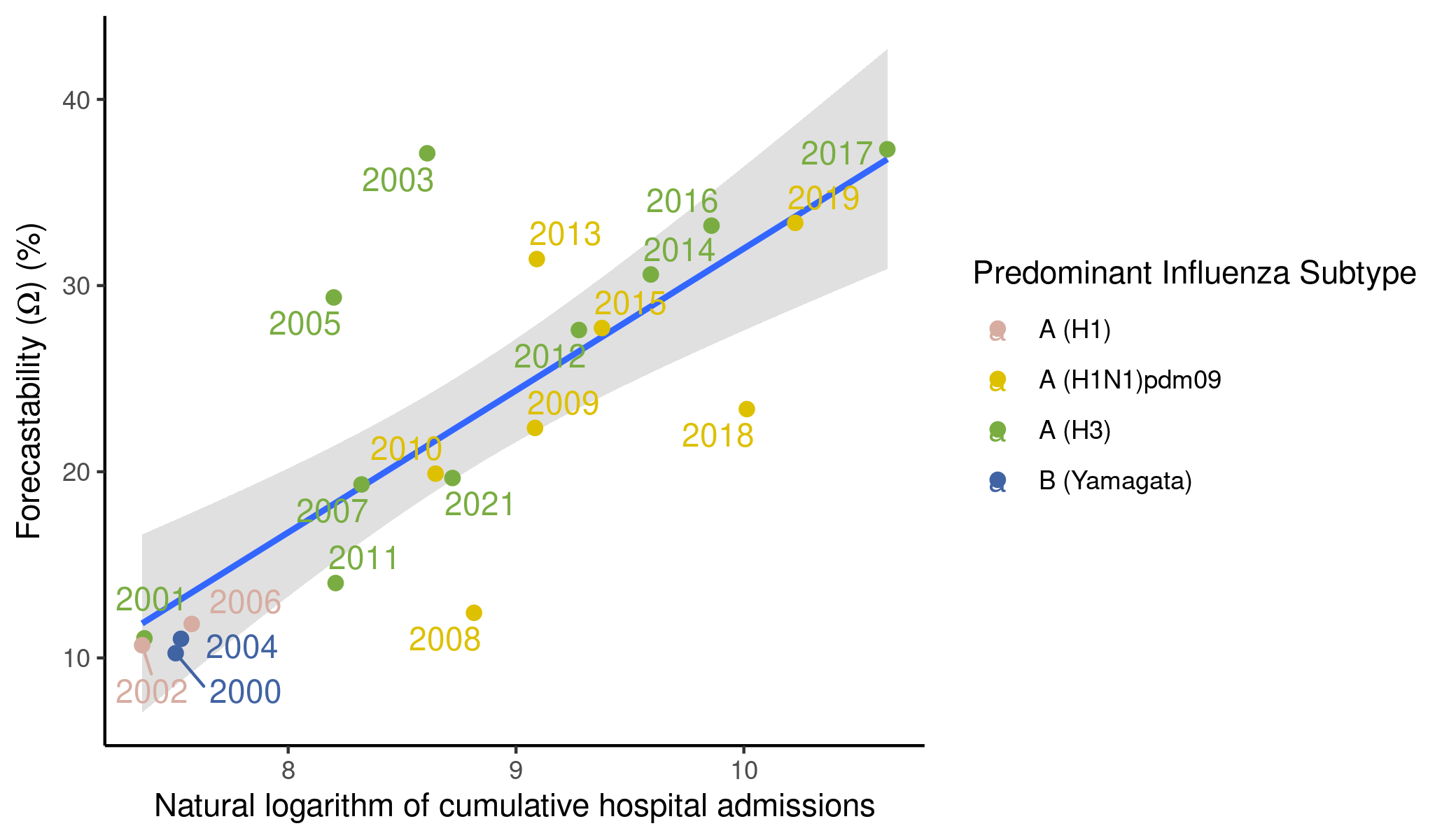

Supplement: S1 Fig — Colors of the points correspond to predominant influenza sub-type as captured by influenza surveillance reports from the California Department of Public Health (CDPH) Influenza Surveillance Program. (TIFF) [file pcbi.1014175.s001.tiff]

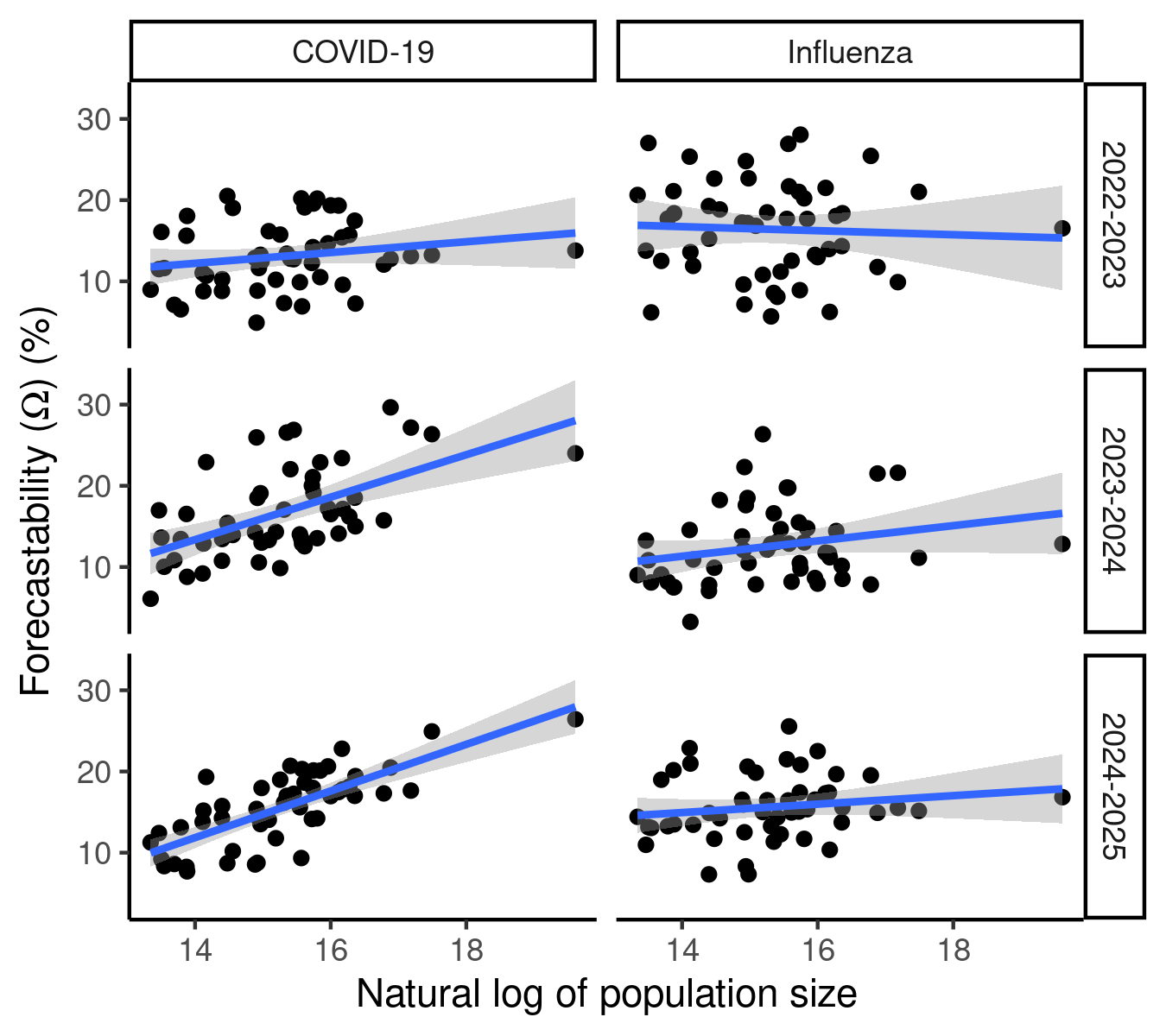

Supplement: S2 Fig — Linear model fit results are described in S3 Table. (TIFF) [file pcbi.1014175.s002.tiff]

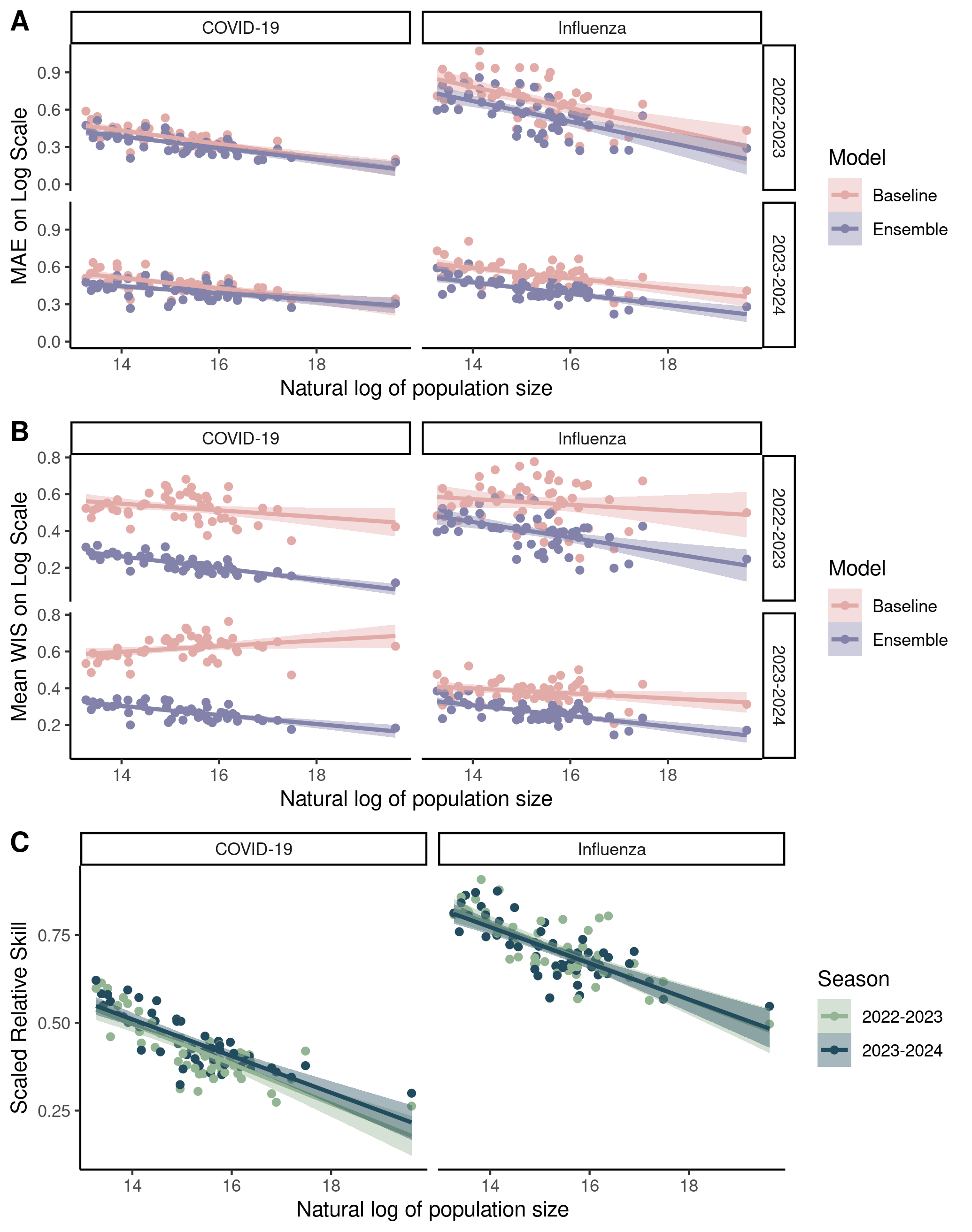

Supplement: S3 Fig — Forecast performance for the baseline and ensemble models as measured by: (A) MAE and (B) mean WIS and (C) scaled relative skill for the ensemble model vs. the natural logarithm of population size. (TIFF) [file pcbi.1014175.s003.tiff]

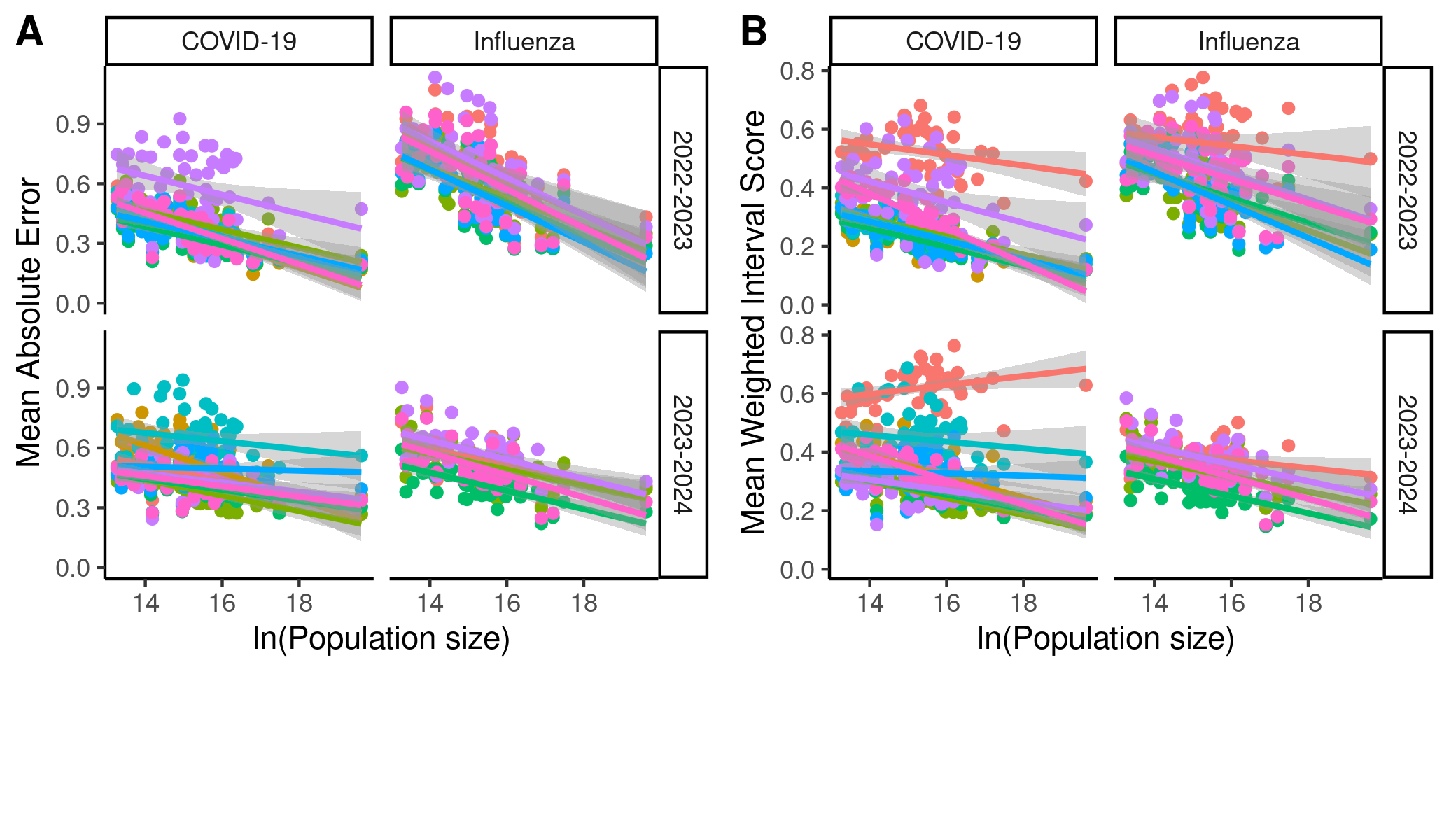

Supplement: S4 Fig — (TIFF) [file pcbi.1014175.s004.tiff]

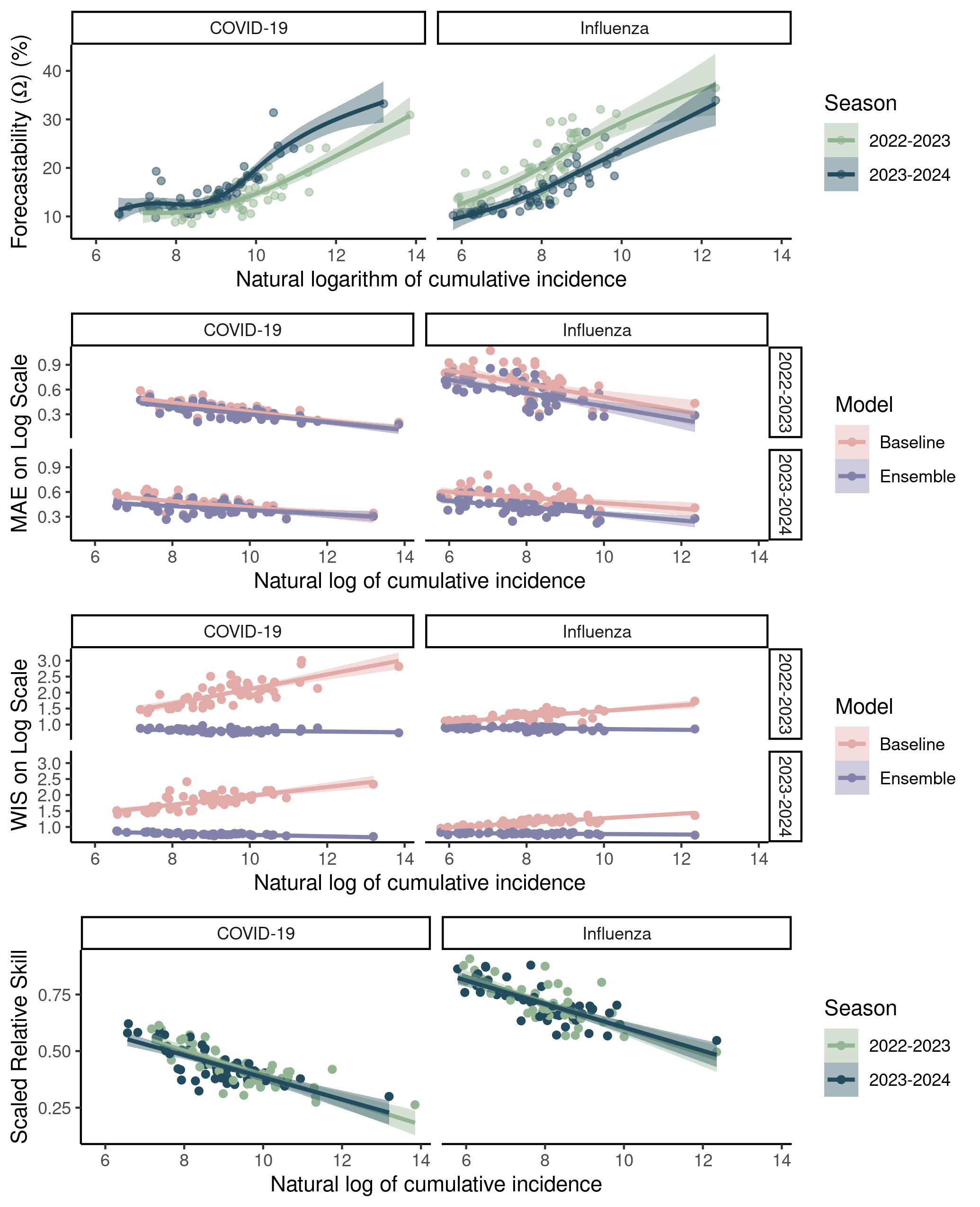

Supplement: S5 Fig — (A) Forecastability (Ω) vs. the natural logarithm of seasonal cumulative incidence. Forecast performance for the baseline and ensemble models as measured by: (B) MAE and (C) mean WIS and (D) scaled relative skill for the ensemble model vs. the natural logarithm of seasonal cumulative incidence. (TIFF) [file pcbi.1014175.s005.tiff]

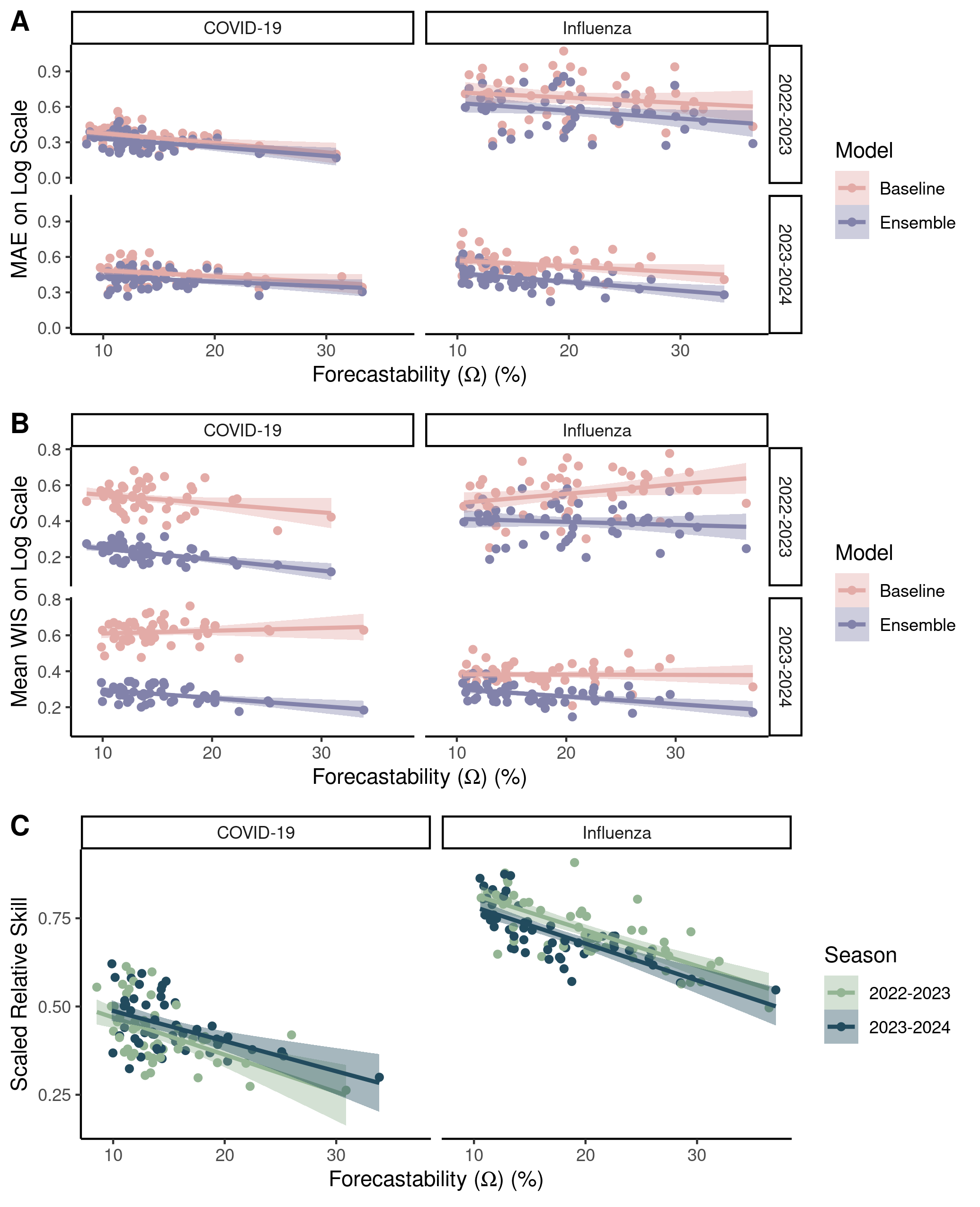

Supplement: S6 Fig — Here seasons are defined as July 1-June 30. (A) Forecastability (Ω) vs. the natural logarithm of population size. Forecast performance for the baseline and ensemble models as measured by: (B) MAE and (C) mean WIS and (D) scaled relative skill for the ensemble model vs. forecastability (Ω). (TIFF) [file pcbi.1014175.s006.tiff]

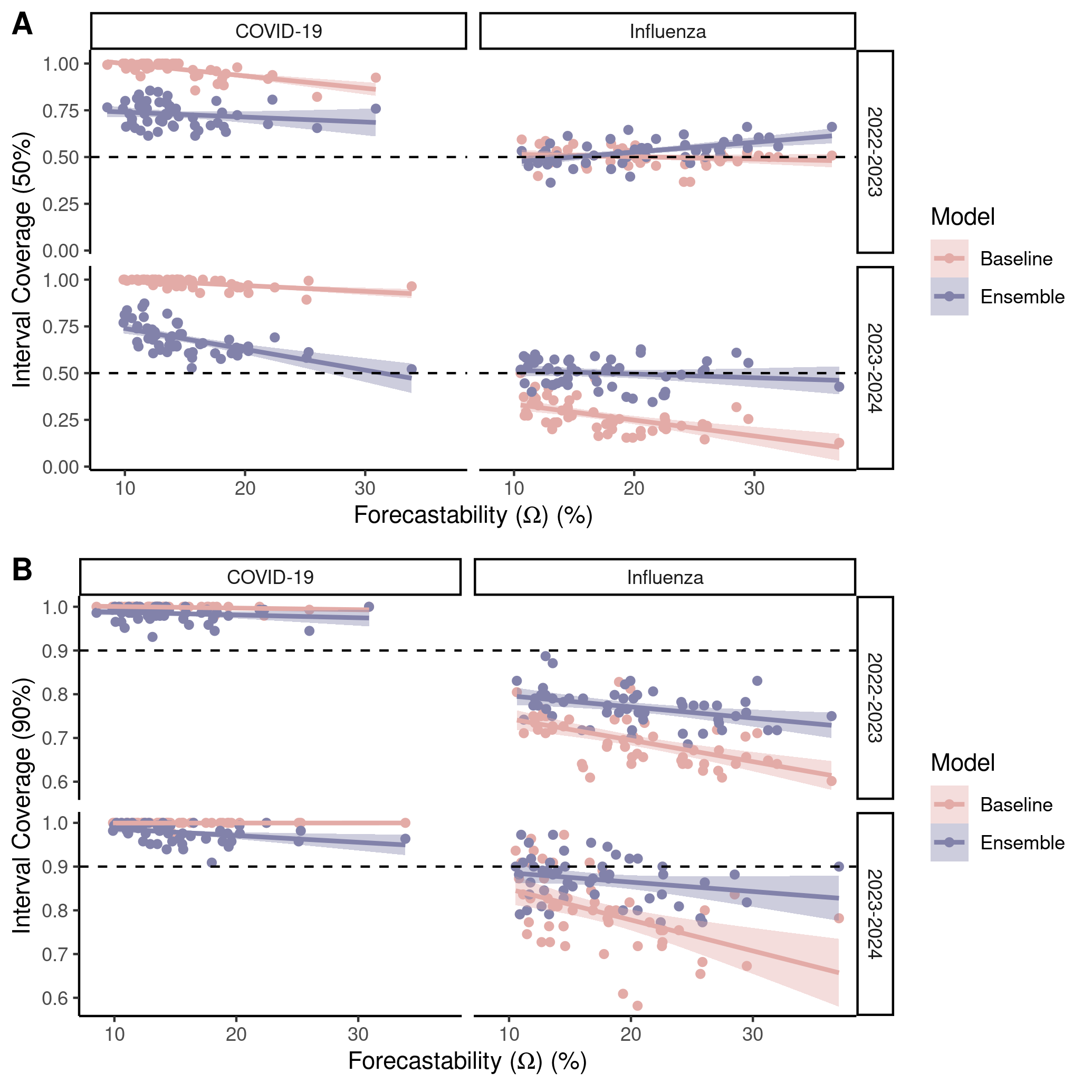

Supplement: S7 Fig — Dashed horizontal lines represent target interval coverage for each panel. Here seasons were classified by the standard influenza season definition of MMWR week 40 to week 39. (TIFF) [file pcbi.1014175.s007.tiff]
