# Supplementary material for: Forecastability of infectious disease time series: are some seasons and pathogens intrinsically more difficult to forecast?
Source: PLoS Comput Biol. 2026 Apr 15;22(4):e1014175. doi: 10.1371/journal.pcbi.1014175 (PMC13102302; doi:10.1371/journal.pcbi.1014175)
Supplement: S1 Table — Values are shown with three significant figures. Bolded rows are those statistically significant at a p = 0.05 threshold. (DOCX) [file pcbi.1014175.s008.docx]

**S1 Table.** Slope estimates from linear model fits for forecastability (Ω) vs. the natural log of population size for %ED visits for COVID-19 and influenza at the U.S. state and national scales as shown in Figure S2. Here seasons were classified by the standard influenza season definition of MMWR week 40 to week 39. Values are shown with three significant figures. Bolded rows are those statistically significant at a p = 0.05 threshold.

| **Metric** | **Disease** | **Season** | **Estimate (beta)** | **Standard error** | **Statistic** | **p-value** |
| --- | --- | --- | --- | --- | --- | --- |
| %ED visits | COVID-19 | 2022-2023 | 0.665 | 0.488 | 1.360 | 1.80e-01 |
| **%ED visits** | **COVID-19** | **2023-2024** | **2.610** | **0.554** | **4.720** | **2.08e-05** |
| **%ED visits** | **COVID-19** | **2024-2025** | **2.870** | **0.367** | **7.820** | **4.09e-10** |
| %ED visits | Influenza | 2022-2023 | -0.249 | 0.716 | -0.347 | 7.30e-01 |
| %ED visits | Influenza | 2023-2024 | 0.942 | 0.557 | 1.690 | 9.74e-02 |
| %ED visits | Influenza | 2024-2025 | 0.521 | 0.472 | 1.100 | 2.75e-01 |
