# Supplementary material for: Forecastability of infectious disease time series: are some seasons and pathogens intrinsically more difficult to forecast?
Source: PLoS Comput Biol. 2026 Apr 15;22(4):e1014175. doi: 10.1371/journal.pcbi.1014175 (PMC13102302; doi:10.1371/journal.pcbi.1014175)
Supplement: S3 Table — Values are shown with three significant figures. Bolded rows are those statistically significant at a p = 0.05 threshold. (DOCX) [file pcbi.1014175.s010.docx]

**S3 Table.** Slope estimates from linear model fits for forecastability (Ω) vs. forecast performance for scaled relative skill of the ensemble model relative to the and baseline model for different forecast horizons targeting laboratory-confirmed (HHS/NHSN) COVID-19 and influenza admissions at the U.S. state and national scales as shown in Fig 5. Values are shown with three significant figures. Bolded rows are those statistically significant at a p = 0.05 threshold.

| **Metric** | **Disease** | **Season** | **Horizon** | **Estimate (beta)** | **Standard error** | **Statistic** | **p-value** |
| --- | --- | --- | --- | --- | --- | --- | --- |
| Scaled Relative Skill | COVID-19 | 2022-2023 | **1** | **-0.0118** | **0.00291** | **-4.04** | **1.84e-4** |
|  |  |  | **2** | **-0.011** | **0.00267** | **-4.11** | **1.45e-4** |
|  |  |  | **3** | **-0.0104** | **0.00249** | **-4.18** | **1.16e-4** |
|  |  |  | **4** | **-0.00853** | **0.00242** | **-3.53** | **9.05e-4** |
|  |  | 2023-2024 | **1** | **-0.00982** | **0.00238** | **-4.12** | **1.42e-4** |
|  |  |  | **2** | **-0.00878** | **0.00219** | **-4.01** | **2.04e-4** |
|  |  |  | **3** | **-0.00822** | **0.00207** | **-3.96** | **2.35e-4** |
|  |  |  | **4** | **-0.0079** | **0.00207** | **-3.83** | **3.63e-4** |
|  | Influenza | 2022-2023 | **1** | **-0.0078** | **0.00144** | **-5.4** | **1.82e-6** |
|  |  |  | **2** | **-0.0104** | **0.00143** | **-7.29** | **2.15e-9** |
|  |  |  | **3** | **-0.0101** | **0.0014** | **-7.21** | **2.85e-9** |
|  |  |  | **4** | **-0.00965** | **0.00144** | **-6.72** | **1.65e-8** |
|  |  | 2023-2024 | **0** | **-0.00873** | **0.00125** | **-6.96** | **6.84e-9** |
|  |  |  | **1** | **-0.0101** | **0.00127** | **-7.98** | **1.81e-10** |
|  |  |  | **2** | **-0.0103** | **0.00146** | **-7.05** | **5.01e-9** |
|  |  |  | **3** | **-0.0111** | **0.00174** | **-6.38** | **5.67e-8** |
