# Supplementary material for: Forecastability of infectious disease time series: are some seasons and pathogens intrinsically more difficult to forecast?
Source: PLoS Comput Biol. 2026 Apr 15;22(4):e1014175. doi: 10.1371/journal.pcbi.1014175 (PMC13102302; doi:10.1371/journal.pcbi.1014175)
Supplement: S4 Table — Here seasons were classified by the standard influenza season definition of MMWR week 40 to week 39. Values are shown with three significant figures. Bolded rows are those statistically significant at a p = 0.05 threshold. (DOCX) [file pcbi.1014175.s011.docx]

**S4 Table.** Slope estimates from linear model fits for forecastability (Ω) vs. forecast interval coverage (at the 50% and 90% levels) for the ensemble and baseline models targeting laboratory-confirmed (HHS/NHSN) COVID-19 and influenza admissions at the U.S. state and national scales as shown in Fig S7. Here seasons were classified by the standard influenza season definition of MMWR week 40 to week 39. Values are shown with three significant figures. Bolded rows are those statistically significant at a p = 0.05 threshold.

| **Metric** | **Disease** | **Model** | **Season** | **Estimate (beta)** | **Standard error** | **Statistic** | **p-value** |
| --- | --- | --- | --- | --- | --- | --- | --- |
| Interval  Coverage  (50%) | COVID-19 | Baseline | **2022-2023** | **-6.59e-03** | **0.001010** | **-6.5500** | **3.03e-08** |
|  |  |  | **2023-2024** | **-3.12e-03** | **0.000619** | **-5.0300** | **6.59e-06** |
|  |  | Ensemble | 2022-2023 | -2.65e-03 | 0.002170 | -1.2200 | 2.27e-01 |
|  |  |  | **2023-2024** | **-1.11e-02** | **0.002040** | **-5.4500** | **1.53e-06** |
|  | Influenza | Baseline | 2022-2023 | -1.27e-03 | 0.001030 | -1.2300 | 2.24e-01 |
|  |  |  | **2023-2024** | **-8.50e-03** | **0.001750** | **-4.8600** | **1.20e-05** |
|  |  | Ensemble | **2022-2023** | **5.26e-03** | **0.001160** | **4.5400** | **3.59e-05** |
|  |  |  | 2023-2024 | -1.99e-03 | 0.001800 | -1.1000 | 2.76e-01 |
| Interval  Coverage  (90%) | COVID-19 | Baseline | **2022-2023** | **-3.65e-04** | **0.000128** | **-2.8700** | **6.06e-03** |
|  |  |  | 2023-2024 | 9.23e-06 | 0.000103 | 0.0897 | 9.29e-01 |
|  |  | Ensemble | 2022-2023 | -6.35e-04 | 0.000558 | -1.1400 | 2.60e-01 |
|  |  |  | 2023-2024 | -1.54e-03 | 0.000604 | -2.5500 | 1.37e-02 |
|  | Influenza | Baseline | **2022-2023** | **-4.91e-03** | **0.000950** | **-5.1600** | **4.21e-06** |
|  |  |  | **2023-2024** | **-7.08e-03** | **0.001870** | **-3.7700** | **4.26e-04** |
|  |  | Ensemble | **2022-2023** | **-2.55e-03** | **0.000840** | **-3.0400** | **3.74e-03** |
|  |  |  | 2023-2024 | -2.15e-03 | 0.001250 | -1.7200 | 9.23e-02 |
