# Supplementary material for: Forecastability of infectious disease time series: are some seasons and pathogens intrinsically more difficult to forecast?
Source: PLoS Comput Biol. 2026 Apr 15;22(4):e1014175. doi: 10.1371/journal.pcbi.1014175 (PMC13102302; doi:10.1371/journal.pcbi.1014175)
Supplement: Attachment — Submitted filename: Response to reviewers 2025_10_29.docx [file pcbi.1014175.s012.docx]

**Response to reviewers for: “Forecastability of infectious disease time series: are some seasons and pathogens intrinsically more difficult to forecast?”**

**(PCOMPBIOL-D-25-01730)**

We thank the editors and reviewers for the thoughtful and constructive comments on our manuscript. We provide point-by-point responses to reviewer comments below and have used “track changes” in the manuscript to highlight revisions. Line numbers are in reference to the clean/“Simple Markup” version in Word. For clarity and to be able to refer to specific comments across sections, we have consecutively numbered editor and reviewer comments. Responses to each comment are bolded below.

As a summary, some key changes that we have made in response to reviewer comments include:

- Revising the Introduction and Discussion to better connect the methodology/results to prior work; specifically, clarifying the relationship between forecastability and existing measures of Shannon, permutation, and spectral entropy
- Adding a simulated Susceptible-Infected-Removed epidemic to Figure 1 as an exemplar time series to build intuition of the forecastability metric (Associate editor comment 0.2)
- Adding example infectious disease time series and their corresponding spectral densities for states with different population sizes (Figure 3B, C)
- Adding forecast coverage as an evaluation metric for forecast performance (Reviewer comment 2.2)
- Displaying forecast performance results as a function of forecast horizon
- Adding an inset in the upper left corner of Figure 2A to zoom in on the lower values of log population size (Reviewer comment 3.10)
- Adding Table 2 with the corresponding slopes and p-values for Figure 4 (Reviewer comment 3.12)
- Expanding upon the Discussion to explore possible mechanisms driving the relationship between forecastability and population size, including looking at the relationship between forecastability and cumulative incidence and looking at forecastability for %ED visits
- Expanding upon the Discussion to suggest possible extensions of this framework for real-time forecasting and evaluation

# Associate Editor:

0.1 I agree with the reviewers that this is an interesting paper around a topic of broad relevance. I also agree that the authors could strengthen their introduction and discussion by better connecting their methodology/results to past work. For example, discussing how your approach is related to permutation entropy, which based on Bandt and Pompe 2002 and Garland et al. 2014 we know is related to more established methods in dynamical systems for assessing predictability. I'm not suggesting a full scale analysis of the relationship, but a conceptual discussion of similarities/differences would help the reader contextualize your work. Additionally, Shao et al. 2024 have a broader review of the topic that transcends permutation entropy.

**Response: Thank you for the specific suggestions for additional literature to support the background and framing of this work (Bandt and Pompe 2002; Garland, James, and Bradley 2014; Shao et al. 2025). We have incorporated these citations into the Introduction to provide a more detailed explanation of the relationship of this metric with Shannon entropy, permutation entropy, and spectral entropy. We have also used this broader scaffolding of entropy theory to reframe our Discussion in the context of prior work.**

0.2 I also agree that it would be helpful to include some additional benchmark time series where we would have a strong a prior assumption about their predictability (e.g., sine waves, Gaussian noise, stochastic SIR simulations).

**Response: We provide examples of some of these benchmark time series already in Figure 1—in particular, Gaussian noise and a sinusoidal function. We have now revised Figure 1 (panels D, E, F) to include a simulated Susceptible Infected Recovered epidemic time series. Per comment #4.3 below, we have also added Figure 3 (panels B &C) to illustrate the relationship between sample time series for different population size targets along with their corresponding power spectra and forecastability scores.**

0.3 The reviewers also raise important points about the choice of window size and the need to better explain the pattern found around population size. If the authors cannot strengthen evidence in favor of a single explanation for the population size effect, they should add to the discussions along the lines pointed out by the reviewers.
**Response:**

- **Regarding comments #2.1 and #4.1 which address window size and applicability for real-time analysis, we have acknowledged that this manuscript is currently best poised for retrospective comparison of seasons and pathogens. We suggest that a rolling window approach for real-time analysis using this metric (or other measures of time series complexity) would be an exciting future direction (lines 525-543).**
- **Per reviewer comments #2.4 & #4.4, we have expanded on the relationship between forecastability and population size by looking at: (1) cumulative seasonal burden vs. forecastability (Figure S5) and (2) looking at the forecastability of an epidemiological signal with a reporting denominator (in this case %ED visits, Figure S2). We have also expanded on our discussion on this question of the effect of population size on this metric (lines 448-465).**

# Reviewer #1:

## Overview

The authors propose an analysis of "forecastability"—defined as the difficulty of predicting a time series—using real-world influenza data. They model forecastability with a combination of spectral density and Shannon entropy, subsequently analyzing the correlation between a series' forecastability and its intrinsic properties, such as population size.

## Positives

1.1 The figures are well-constructed and clearly presented, effectively illustrating the varying results across different subgroups.
1.2 The authors provide a comprehensive literature review of related work.
1.3 The paper includes a number of experiments and corresponding analyses, which contribute considerable information to the topic.

**Response 1.1-1.3: Thank you for your positive feedback. We are glad that you found the figures, literature review, and analyses valuable.**

## Weaknesses

1.4 The introduction is poorly organized and contains extraneous information. For instance, the authors dedicate long paragraphs to conclusions from prior work on forecastability and uncertainty (e.g., lines 68-77, 83-89) without clearly connecting these findings to their own proposed methodology.

**Response: Per the Associate Editor’s comment #0.1 above, we have strengthened the introduction by clarifying connection to prior work, particularly around the relationship between permutation entropy and spectral entropy. We have further clarified what has already been done in prior work and how this connects to the present study.**

1.5 The insights inside the designing of the forecastability metric are unclear. The authors should provide more detailed intuitions on why the differential entropy of Fourier-form spectral density would provide insight to analyzing the forecastability.

**Response:** **We have clarified that are using a pre-existing metric of time series complexity based in spectral entropy called “forecastability” that was defined by Goerg (2013). We have added a brief intuition spectral entropy may provide insight into future predictability (lines 109-114): “A time series with high spectral entropy (i.e., low forecastability) suggests that the power spectrum of the signal is spread across multiple frequencies, which is indicative of chaotic behavior with less structure to exploit for prediction. In contrast, a time series with low spectral entropy or high forecastability will have its power signal concentrated in a few frequencies, which points to periodic behavior or seasonality that may be leveraged for forecasting.”**

1.6 Following the previous point, I doubt the design of this metric. The whole metric is calculated based on the autocovariance function, which cannot reflect the predictability in many cases – for example, in sequences where non-linear patterns (like quadratic or other complex patterns) are dominant, autocovariance will not be accurate in the sense of predictability.

**Response: We included the explanation of the autocovariance to help build intuition of this metric, but spectral entropy is better poised to reflect nonlinear and nonstationary dynamics than time-domain metrics like autocovariance.**

**We do, however, acknowledge that this is still only one potential measure of time series complexity in the Discussion section (lines 525-527): “There are numerous other ways to measure time series complexity and entropy (Kang, Hyndman, and Smith-Miles 2016; Papacharalampous et al. 2022), such as permutation entropy (Garland, James, and Bradley 2014; Mills et al. 2025; Scarpino and Petri 2019), and future work could compare other metrics that might capture other patterns or features of non-linearity important to an infectious disease context.”**

1.7 The methodology lacks detail regarding the temporal relationship between the forecastability calculation and the forecasting procedure. It is unclear whether the forecastability metrics are calculated using the test set (i.e., ground truth data) or only historical data available prior to the prediction window. Can this forecastability metric be determined before accessing the prediction window?

**Response: In our current implementation, we are calculating the forecastability for an entire respiratory virus season at a time. Per comment 1.11 below, this presupposes complete data/knowledge for the prediction window. It would also be possible to calculate forecastability on a rolling window using versioned data (as suggested by Reviewer 2 below). We have further clarified the temporal relationship between forecastability calculation and forecasting procedure in the Methods section:**

- **“Forecastability scores were calculated for each location (i.e., counties for HCAI data or states and national U.S. for NHSN) and for each respiratory virus season as defined in the “Data” section above” (lines 216-218).**
- **“MAE, WIS, scaled relative skill, and interval coverage were calculated for each location, target date, and model combination across one-, two-, three-, and four-week horizons using the scoringutils package in R (Bosse et al. 2022). To explore a potential relationship with forecastability, each metric (i.e. MAE, WIS, scaled relative skill) was summarized by taking the mean value for each combination of target location and respiratory virus season” (lines 265-269).**
  1.8 The authors are calculating model-agnostic "forecastability", but (understandably) evaluating their results with model-based prediction results. However, this means that there should be more details and discussions around the selection of model architectures, instead of simply calling them “baseline” and “sota” models.

**Response: We’re not entirely clear on what “sota” models was meant to refer to here—we don’t use the phrase “sota” or “state of the art” anywhere in the manuscript. The Hub methodologies for generating baseline and ensemble models from submissions are well documented. We now provide more context as follows:**

- **The constituent types of models that are submitted to the COVID-19 and FluSight Forecast hubs (lines 48-50): “Individual model types vary widely in their formulation and may include mechanistic (e.g., Susceptible-Infected-Recovered [SIR] compartmental models), statistical, machine learning, or ensemble approaches (Cramer, Ray, et al. 2022; Mathis et al. 2024).”**
- **The underlying architecture for the ensemble and baseline models commonly used in these infectious disease forecasting contexts (lines 176-182): (Cramer, Huang, et al. 2022; Mathis et al. 2024). “Specifically, we focused on a comparison of baseline and ensemble models, which have well documented methodologies from these two hubs. Briefly, the ensemble model takes the unweighted median of each quantile for all eligible forecasts submitted to the hub for a given target and date (Cramer, Huang, et al. 2022; Mathis et al. 2024). The baseline model is used as a neutral comparison model with the median value of future forecasts equal to the prior week’s incidence and uncertainty defined by historical changes in weekly incidence of the time series (Cramer, Huang, et al. 2022; Mathis et al. 2024).”**

## Points for improvement

1.9 The authors should clarify their contributions and clearly state the relationship between their work and prior work. Plus, in the methodology, it’s hard to tell whether a metric is novel and was developed by the authors or inherited from previous work.

**Response:** **Per comment 1.4 above, we have clarified our contributions and stated the relationship between our work and prior studies. We have also more clearly highlighted in the Methods section that the forecastability metric is taken from prior work conducted by (Goerg 2013), but applied specifically to this context of infectious disease forecasting in this manuscript (lines 187-190): “In this manuscript, we use a metric of time series complexity based in spectral entropy called “forecastability” that was defined previously by Goerg (2013). We provide a brief overview of the concepts central to its definition, but refer the interested reader to Goerg (2013) for a more detailed derivation.”**

1.10 More theoretical analysis has to be done for the forecastability metric. Theoretical results like some lemmas or theorems regarding any nice properties of the calculated metric would help people understand the metric better. For example, a lemma illustrating “the calculated metric would best reflect the predictability for sequences under xxx condition (seasonality, linear relationship, etc.)” would be helpful.

**Response: The forecastability metric we use for this analysis here has already been outlined in detail (Goerg 2013). We refer the Reviewer to this body of work for a more detailed derivation. Per comment 1.9 above, we have aimed to further emphasize that this is an already developed metric that we are applying in a new domain context.**

1.11 The authors should be cautious about the input and output time range of time-series. Calculating predictability based on historical data or current data will form completely different stories.

**Response: This is an important point that we address in the Discussion (lines 527-528): “One key limitation of this analysis is that we measured the forecastability of an entire season, while forecast metrics presented here represent mean scores of numerous individual forecasts through time—each occurring at time points with imperfect or incomplete information for the season up to that given date.” We have added a note in the Discussion addressing the potential importance versioned data when calculating this metric (lines 531-533): “This retrospective approach also means that we did not account for versioning of the underlying target data, which is a process that modelers must contend with when generating forecasts in real time.”**

1.12 The authors should formulate the equations better and double-check for typos. For example, in the equation inside line 155, the authors mentioned x_{t-k}, while no explanations are provided around k. Should this be x_{t-h} instead?

**Response: Thank you for catching the typo in our equation—we have corrected the mistake (line 194) and have done our best to double check our equation formulations throughout the text.**

# Reviewer #2:

The manuscript investigates whether properties of epidemiological time series help explain variation in forecasting performance across diseases, locations, and seasons. It estimates “forecastability” for one state data stream that includes county-level and two national data streams at the state level. The paper relates the forecastability metric for the two national data streams to forecast accuracy for laboratory-confirmed hospital admissions of COVID-19 and influenza using archived forecasts from the COVID-19 Forecast Hub and FluSight. It examines how forecastability varies with population size and season burden and explores sensitivity to reporting frequency and smoothing. The article is well written. Specific recommendations/comments follow:

2.1 My major concern is with the framing of the paper. It seems like the authors could be proposing this metric of “forecastability” as a way to help public health officials and forecasting teams evaluate the potential utility of various public health data streams for seasonal pathogen forecasting or for identifying what sub-state geographic levels have enough data to support forecasting since this metric appears to help identify indicators that may be more noise than signal. However, I’m not sure I follow the practical Interpretability of the forecastability metric to help understand the “ease of forecasting” a signal since it seems to indicate that seasons that have been historically been more severe for influenza would be the ones “easiest” to forecast (e.g. 2017/18). It appears the metric is identifying seasons with large, single peaks, which does not mean they are easier to forecast (a fact we know from forecast performance during seasons like 2017/18), and we don’t know what type of season we’re having when you’re forecasting. Are there ways the authors could help us understand the real-time applicability of this metric. Could the authors calculate forecastability on rolling windows for example?

**Response: We appreciate your concern regarding the framing of the paper. As a first approximation, this manuscript would be best poised for retrospective comparison of seasons and pathogens; the analysis is meant as a step in the direction proposed by the reviewers but does not ultimately resolve the tension pointed out of how the forecastability metric might be used in real-time.**

**We acknowledge the rolling window approach as an intriguing future direction in the Discussion (lines 533-538): “Future analyses could explore applications for real-time forecasting applications by applying this metric to versioned data and exploring its sensitivity to a rolling window. Longer rolling windows, containing multiple years, might be able to better illuminate differences in periodicity for pathogens with annual cycles. It would also be interesting to see if the relationship between forecastability and forecast performance changes during periods of the epidemic, e.g., near the peak or during low levels of activity.”**

**We examined the relationship between season peak and cumulative burden for historical influenza seasons using non-laboratory confirmed hospitalizations for California. We would need to use a different target like ILI to be able more directly tie these findings to forecast performance for historical seasons prior to 2022-2023. One thing to note is that the forecastability metric is scale invariant, and so we believe this metric is capturing something about the fundamental shape of the time series, not just reflecting the absolute magnitude or cumulative burden. We’ve added a brief note on this point to the Discussion (lines 443-447): “However, it is worth noting that the forecastability metric is scale invariant (Goerg 2013), i.e., for a given time series, the forecastability score for the raw number of weekly admissions and the weekly admissions per 100K would be the same. We therefore believe that this metric reflects something more about the shape of the season other than just the absolute magnitude of the burden itself.”**

2.2 The authors don’t provide any comparisons to forecast coverage. This seems like it could be a useful addition to see if the findings are consistent with that accuracy metric.

**Response: We have added Figure S7 and Table S4 to explore the relationship between forecastability and forecast interval coverage at the 50% and 90% levels. We have added a description of interval coverage in the Methods (lines 249-253) and discussed these findings in the Results section (lines 322-330). The relationship between forecastability and forecast interval coverage is more ambiguous than for MAE, WIS, and relative WIS.**

2.3 The first part of the analysis (comparing CA county- and state-level data for syndromic influenza admissions) doesn’t exactly seem to fit with the rest of the analysis, and the comparison to flu types in this section feels a bit like a tangent. I’m not sure exactly clear what this part is adding to the manuscript.

**Response: The goal of this part of the analysis was twofold: (1) to take advantage of a different metric (non lab-confirmed influenza hospitalizations) for which we had longer/more consistent time series data and multiple respiratory virus seasons for comparison (~2000-2022); and (2) look at the forecastability metric across a range of scales (county vs. state), as compared to state vs. national for NHSN data set. The NHSN data set is comparatively new with mandatory reporting only beginning as of February 2022 and so is limited in duration.**

2.4 It seems difficult to parse out higher population states vs. states having more data (e.g. you would expect a time series of hospitalizations for influenza or COVID-19 to have more reported hospitalizations/higher peak hospitalizations from larger populations and could have higher forecastability with this metric). Is there a way for the authors to explore this since I’m not sure their conclusions about forecastability being associated with population size are robust since it seems having more data could explain that. Could the authors look at data like % ED visits for a pathogen since that may be less directly impacted by the population (as long as ED coverage was robust).

**Response: We thank the reviewer for this valuable point. Since recent forecasting hub efforts have focused on number of weekly hospital admissions, we focused on this as the primary outcome metric. We do believe that with mandatory reporting in place that NHSN hospitalizations represent a reasonable gold standard in terms of coverage and reporting across the US** (Frutos et al. 2025; Scobie et al. 2023)**.**

**With respect to adjusting for more populous states having more data or higher reporting, we also investigated the relationships between (1) cumulative seasonal burden and forecastability and (2) cumulative seasonal burden and forecast performance—both of which remain robust and show similar patterns to forecastability vs. state population size (Figure S5).**

**We briefly looked at the relationship between %ED visits and forecastability and observed more ambiguous trends related to population size, as hypothesized by the reviewer (Figure S2, Table S1). Only COVID-19 %ED visits for the 2023-2024 and 2024-2025 seasons displayed a significant positive relationship between forecastability and population size. %ED visits are now an official target for the FluSight Forecasting Challenge and COVID Forecasting Hub for the 2025-2026 season, so future work could compare forecast performance and forecastability for this new target.**

**With these two additional sub-analyses, we believe that part of the point is that larger population sizes (without some sort of denominator) will have less noise in the time series and thus have higher forecastability, again noting this observation from prior forecasting hub evaluations** (Lopez et al. 2024; White et al. 2023)**. We do not believe that anything biological is necessarily going on related to the somewhat arbitrary political boundaries that divide up US states into different catchment areas.**

**We have expanded upon these points in the Discussion (lines 448-471): “Our results also echoed prior work that found a relationship between population size of the forecast target and forecast performance (Fig S3 & S4) (Lopez et al. 2024; White et al. 2023). This observed relationship between forecastability and population size potentially reflects general intuition, i.e., that larger populations should have signals closer to the mean field, with corresponding weaker high frequency components, resulting in a higher forecastability score. However, a key assumption of this conclusion is that disease incidence or burden generally scales with population size. To test this assumption, we assessed the relationship between cumulative seasonal incidence, forecastability, and forecast performance. Forecastability increased and forecast performance improved with higher cumulative seasonal incidence (Figure S5). To further investigate a possible mechanism for this pattern, we also looked at the relationship between population size and forecastability for %ED visits, which has an explicit denominator that accounts for volume of reporting. In this case, the relationship between populations size and forecastability was more equivocal with only COVID-19 %ED visits for the 2023-2024 & 2024-2025 seasons displaying significant positive relationships. Since we might expect that a target metric with a denominator might create smoother time series overall regardless of total incidence, a more subtle relationship between population size and forecastability was not surprising… Together these findings point to an improved signal-to-noise ratio arising from data aggregation—whether spatial (i.e., larger populations with larger reporting volume) or temporal—as a potential mechanism for a higher forecastability score, rather than some distinct transmission phenomenon occurring at specific scales.”**

2.5 The paper states that forecasting performance “generally improved with higher forecastability when controlling for population size,” but the main model fits reported (Table 1) appear to regress performance on forecastability (per disease and season), without anything explicitly included on population. What analysis are controlling for population?

**Response: Instead of using population as an offset in the regression, we mitigate the effects of population size by taking the natural log of the weighted interval score (WIS) when scoring forecast performance. Per Reviewer Comment #3.3 below, we have softened the language throughout the manuscript to indicate that this approach may “mitigate the effects” rather than “control for” differences in population size across target locations.**

2.6 It would be helpful if the authors provided some examples of the proposed frameworks to adjust scores over seasons to understand what additional information would be gleaned by doing what the authors recommend in this part: “This raises the question of whether forecasting challenges should also be: (1) controlling for some version of signal predictability based on geographic scale or (2) controlling for season predictability post-hoc when scoring forecasts.”

**Response: We have added that a regression framework could help standardize scoring across seasons (lines 504-508): “For example, it might be possible to standardize or scale forecast scores across seasons using a regression framework where one of the covariates is a forecastability score (or time series complexity metric). This could help explain whether variability in model performance reflects the proclivity of individual model types to better handle certain regimes or patterns of time series complexity (Garland, James, and Bradley 2014).”**

# Reviewer #3:

The authors investigate how a time series evaluation metric of predictability, forecastability, changes across locations with different population sizes as well as across both COVID-19 and influenza hospital admissions for multiple seasons. This is an important contribution to the field, particularly as more forecasting efforts are being undertaken at more local geographic scale. The findings of this manuscript suggest that these smaller locations may have less predictable infectious disease time series, perhaps indicating that there may be limits to the utility of forecasting at such scales. The manuscript is well written and addressed many of my questions shortly after they were raised. This is further supported by the observation that forecasting performance generally improved for time series with higher forecastability scores, even when controlling for population size (although see note below about controlling for population size vs. disease incidence). The result that some respiratory virus seasons were more “forecastable” than others and the observed positive relationship between forecastability and cumulative burden and peak burden is also interesting.

3.1 The role of the smoothed time series results analysis could be strengthened. It doesn’t seem that this directly relates to the claim that “larger counts leading to smoother time series” may increase forecastability. It’s not immediately clear what impacts this would have for real-time forecasting. Is there an implied relationship between the amount of smoothing and the extent of aggregation (e.g., daily vs. weekly)? I also found it a bit difficult to reconcile these results in lines 260-269. There is some discussion around these results in lines 340-345 concluding with “we were not able to tie these observation directly to forecast performance”. How should the reader interpret these results in whether it might be better or worse to have forecast challenges on weekly vs daily data? Or since the observation cannot be tied to forecast performance, is it not possible to provide support for one versus the other.

**Response: Thank you for the comment. For clarity, we have separated the issues of time series aggregation and down sampling in the Discussion.** **We do believe that aggregation (whether temporal or spatial) that improves the signal-to-noise ratio is the primary mechanism by which we see higher forecastability scores for more populous states (Fig 3), states with higher cumulative burden (Fig S5), or time series smoothed with a longer rolling window (Fig 7).**

**We also provided a more detailed interpretation of the downsampling results as it relates to spectral entropy. Although we lack information on underlying training data that would allow us to directly tie forecast performance to reporting frequency, we do further speculate on the implications for forecasting challenges on weekly vs. daily data (lines 473-486): “…this particular metric of forecastability appears to be strongly affected by data frequency (e.g., daily vs. weekly); this makes sense for a metric based in spectral entropy where changes in reporting frequency can change the resulting frequency content…We could not tie these observations directly to forecast performance because of a lack of information about which models were trained on daily vs. weekly data…Nevertheless, this pattern suggests that information with the potential to improve forecasting performance may be lost when switching from daily to weekly reporting frequency.”**

3.2 Would it be of interest to see how forecasting performance changed when not controlling for population size? It seems that these results would likely further confirm the results of the manuscript. Perhaps this is out of scope, but I do think there may be interesting differences between evaluating forecasting performance on transformed and untransformed time series.

**Response: We did not include the results in the manuscript, but we did originally perform this analysis with raw, untransformed WIS and did not see any relationship between forecastability and population size. Since WIS scales with absolute magnitude of the target and since we include very large range in population sizes across different orders of magnitude (e.g., 500,000 for Wyoming vs. 340 million for US)—any improvement in forecast performance was offset by the increase in magnitude of the disease burden.**

3.3 It may be important to state that the log transformation in this analysis mitigates the impact of different sized targets, but I’m not sure that it exactly gives equal weight to location targets with different sizes as stated in lines 60-61. Suggest softening the language here and elsewhere (e.g. 109-110) to something more like “mitigates the impact of different sized targets”. Also since the log-transformation is performed on the target itself and is not a per capita transformation, this makes different locations more comparable but doesn’t fully “control for population size” as implied in the abstract and throughout (e.g., line 318 “when controlling for population size”). It may also be possible to address this as a caveat in the discussion section, by making the assumption that disease incidence (generally) tends to scale with population size.

**Response: Thank you for catching this. We have softened the language to reflect that the log transformation of WIS scoring mitigates the impact of different sized targets but does not fully control for the effects of population size (e.g., lines 21, 35, 151, 259, 415, 502). We have also added a caveat in the Discussion section regarding the assumption that disease incidence tends to scale with population size (lines 454-455): “…a key assumption of this conclusion is that disease incidence general scales with population size.”**

3.4 Lines 68-70. It’s interesting that predictability decreased with increasing time series length, as generally forecasters are interested in having more data to fit their models.

**Response: We agree! And this may be a fundamental limitation of these entropy-based approaches—longer time series are (on average) going to get messier (e.g., different periodicity, different peak magnitudes, etc. across seasons), which means that by default forecastability will likely decline. This is a counterintuitive result in the context of forecasting where more historical data is usually a boon.**

3.5 Line 147-148. Comment that ensembles generally provide the most robust performance seems more fitting for discussion or background than methods.

**Response: We have removed this point from the Methods and now reference this briefly in the introduction (lines 50-51): “…individual models of all types historically have failed to outperform hub ensembles consistently through time.”**

3.6 Line 168-169. Is there a reference for the definition of “forecastability” that should also be included here?

**Response: We have moved the relevant reference (Goerg 2013) from the end of the sentence to before the equation on line 204.**

3.7 Line 214. “relative skill” instead of “relative risk”?

**Response: Thanks for catching this. We have corrected the term to “relative skill” instead of “relative risk” (line 265).**

3.8 Line 243. “(Fig 3B-D)” consider also referencing Table 1 which contains information about which relationships were statistically significant.

**Response: To the end of the caption for Figure 4 (formerly Figure 3B-D), we have added the following reference to Table 1 (line 367-368): “Table 1 reports the regression fits for panels A-C, as well as their statistical significance.”**

3.9 Line 244. “Except for the 2022-2023 influenza season, …” Would it be possible to comment in the discussion as to whether there are characteristics of the 2022-2023 season that may have contributed to MAE and WIS not decreasing with higher forecastability?

**Response: We have added a brief discussion on characteristics of the 2022-2023 influenza season that may have contributed to MAE and WIS not decreasing with higher forecastability (lines 425-433): “The exception to this pattern was the 2022-2023 influenza season pattern where MAE and WIS scores did not covary significantly with forecastability (Fig 3B, 3C, Table 2). This mismatch in the ensemble performance may have been caused by irregular influenza dynamics in the aftermath of the COVID-19 pandemic (Borchering et al. 2024). During this period, the United States experienced an unusual increase in influenza activity during the summer months of 2022, as well as a shift to forecasting laboratory-confirmed hospital admissions beginning in 2022 for the CDC FluSight Forecasting Challenge instead of influenza-like illness (Mathis et al. 2024). It also highlights that forecasting a completely new signal remains challenging regardless of the features of the time series.”**

3.10 Figure 2A. It might be nice to have an inset in the upper left corner that zooms in for the lower values of log population size.

**Response: We have added an inset in the upper left corner of Figure 2A to display the lower range of log population size.**

3.11 Figure 2B. Is 2020-2021 also excluded here?

**Response: Yes, 2020-2021 was also excluded for the analysis shown in Figure 2B. We have added a second parenthetical (line 351) to the figure caption to this effect.**

3.12 Figure 4. It would be helpful to add a table with the slopes and p-values (as was done for Figure 3 in Table 1) to be able to highlight which of the displayed relationships are significant. Depending on how many relationships are significant or not, you could also consider mentioning this in the text or Figure caption.

**Response: We have added a Table 2 with the slopes and p-values for Figure 6 (formerly Figure 4), and mentioned significant relationships in the Results text and figure caption (lines 382-384): “While all combinations of disease and season were statistically significant for daily reporting, only COVID-19 for the 2022-2023 had a significant relationship between population size and forecastability for weekly cadence (Table 2).”**

3.13 Table 1. I think “Figure 2” should be “Figure 3”.

**Response: Thanks for catching this mismatch. We have corrected the reference from “Figure 2” to “Fig 4” in the Table 1 caption (line 398).**

3.14 Line 313-314. Suggest clarifying whether observed differences in measured forecastability correlated with resulting forecast performance of the ensemble forecasts? vs. baseline forecasts or both.

**Response: We have further clarified that these differences in performance were observed for the ensemble but not for corresponding baseline models (lines 414-419): “…ensemble forecast performance improves for time series with higher forecastability scores …Scaled relative skill of the ensemble against the baseline model improved with increasing forecastability … and these gains came from improvements in ensemble performance rather than changes in the baseline model performance, which remained relatively invariant to the time series’ forecastability.”**

3.15 Line 351-353. Agree that the shift to forecasting a new signal is difficult. Suggest editing to say something like “(e.g., the shift to forecasting laboratory-confirmed hospital admissions beginning in 2022 for FluSight)”. Since there were technically FluSight forecasts for the latter part of the 2021-2022 season.
**Response: We have restructured the Discussion so that we now devote a single paragraph to potential discrepancies in the 2022-2023 influenza season, including a clarification on the changes in reporting (lines 428-433): “…During this period, the United States experienced an unusual increase in influenza activity during the summer months of 2022, as well as a shift to forecasting laboratory-confirmed hospital admissions beginning in 2022 for the CDC FluSight Forecasting Challenge instead of influenza-like illness** (Mathis et al. 2024)**. It also highlights that forecasting a completely new signal remains challenging regardless of the features of the time series.”**

3.16 Line 354-355. I appreciate that you discuss how this is just one metric later in the discussion. You could hint that it is important to keep in mind that the “second notable pattern” result here is for this particular definition of forecastability.
**Response: We have edited this sentence accordingly (lines 487-488): “Another notable pattern for this particular metric of forecastability is that there is a consistent plateau at about 10% for smaller geographies…”**

3.17 Line 358-360. 1 and 2 are really dependent on the goal. As mentioned, the latter really is more important when trying to compare across seasons. Do you have any suggestions to include for handling 1 and what would be the explicit goals? It seems like if the forecasts are less predictable for smaller geographic scales then it makes sense that the scores would indicate that difference in performance.
**Response: Since metrics like MAE and WIS scale with absolute magnitude of the target, it would be important to at least also look at evaluation on some sort of transformed scale and/or include some sort of relative error metric in evaluation. Many formal evaluations already do this, but it seemed worth emphasizing-- otherwise, these differences across geography can be obscured or deemphasized. We’ve added the following clarification (lines 501-503): “The former can be addressed by including some form of relative error metric or transformed scoring that mitigates the absolute magnitude of target size (Bosse et al. 2023).”**

3.18 Lines 362-364. I agree completely. You could also consider mentioning that the inherent difficulty of forecasting for smaller geographies would suggest that forecasts for smaller geographies should be communicated with this disclaimer. I wonder if it would be worth mentioning the caveat that some “local level” forecasts could be for populations of larger size (e.g., NYC) and may not be subject to the same constraints of limited population size for other local level forecasts.
**Response: This is a good point to make for public health implementation. We have added the following text (lines 491-496): “Reduced model performance at smaller population size suggests that communication of these forecast results to public health practitioners may need an explicit disclaimer. However, this consideration may not apply to sub-state “local level” forecasts of larger population size, e.g., large metropolises like New York City. Since the forecastability scores of % ED visits were generally less dependent on population size (Fig S2), using a metric with an implicit denominator for reporting volume may also help mitigate these tradeoffs.”**

3.19 Lines 386-387. suggesting that this metric is “not capturing all features relevant to forecasting performance” is an interesting observation.
**Response: We agree! We think this metric is just one possible way to measure time series complexity. We’ve expanded a bit more on this point in the Discussion (lines 525-527): “There are numerous other ways to measure time series complexity and entropy (Kang, Hyndman, and Smith-Miles 2016; Papacharalampous et al. 2022), such as permutation entropy (Garland, James, and Bradley 2014; Mills et al. 2025; Scarpino and Petri 2019), and future work could compare other metrics that might capture other patterns or features of non-linearity important to an infectious disease context.”**

3.20 Lines 389-390. “… how forecasts contribute to decision making at different thresholds of uncertainty and predictability” I’m not sure that this directly follows. Perhaps a slight clarification to “… how forecasts should contribute to decision making…”
**Response: We have made the suggested change in wording for clarification (line 561).**

3.21 Data availability section. It may be worth also providing the updated links to where the data can be found on data.cdc.gov (since I believe the healthdata.gov sites no longer provide the data).
**Response: Thank you for flagging. We double checked, and these links still provide stable APIs/data exports for NHSN state-level and facility level data for the analysis period of interest in our manuscript up through the 2023-2024 season.**

3.22 Reference #5. It may be worth updating this medRxiv reference to the final Nature Communications version: Mathis SM, Webber AE, León TM, Murray EL, Sun M, White LA, et al. Evaluation of FluSight influenza forecasting in the 2021–22 and 2022–23 seasons with a new target laboratory-confirmed influenza hospitalizations. Nature Communications. 2024 Jul 26;15(1):6289.

**Response: Thank you for catching this. We have updated this reference to reflect the information of the final version as published in *Nature Communications* (line 626-628)*.***

# Reviewer #4:

I enjoyed this paper, which is straightforward and well presented. It ties in measures of time series predictability (ie, repeated cycles in the data summarized by spectral density and entropy) with prediction performances. Authors use short-term forecasts for influenza and COVID-19 across US jurisdictions as case studies and highlight differences between pathogens, seasons and population sizes.

I have a few primary questions and additional comments that are more focused on interpretation.

Main comments:
4.1 I was a bit perplexed by the spectral/entropy analysis (ie forecastibility estimate) of flu and COVID-19 data by season. As I understand, this is a spectral analysis of 1-yr chunks of data (based on the R code nicely provided on github). I agree it would be great to understand variability in predictability between seasons, but what does a 1-yr analysis mean for a disease that primarily has an annual cycle? Wouldn’t it be more epidemiologically relevant to run these analyses using moving windows, where each window includes several years, and identify periods that are more or less predictable?

**Response: It would certainly be interesting to look at a rolling window analysis. One challenge or limitation with infectious disease data is the lack of overlap between (1) long historical infectious disease time series; (2) similar reporting mechanisms across diseases; and (3) corresponding forecasts for target diseases. For example, COVID-19 is of course a still relatively “new” pathogen. If we rely on NHSN data which has comparable reporting for influenza and COVID-19, mandated reporting of influenza hospital admissions didn’t begin until February 2022 and reporting became voluntary in the summer of 2024, which means that we lack the ability to use longer, multi-year rolling windows since we have chosen to emphasize the COVID-19 vs. influenza comparison.**

**We have emphasized this as a potential future direction in the Discussion (lines 533-536): “Future analyses could explore applications for real-time forecasting applications by applying this metric to versioned data and exploring its sensitivity to a rolling window. Longer rolling windows, containing multiple years, might be able to better illuminate differences in periodicity for pathogens with annual cycles.”**

4.2 Relatedly, I wondered whether the analysis of daily vs weekly aggregation is telling us primarily about the forecastability of the reporting process rather than disease dynamics. With the daily data, there are very highly predictable cycles in the data, which are the WE reporting effects, but have potentially very little to do with transmission.
**Response: This is a valid concern, but we do not believe that is a mechanism at play here because hospital admission time series were converted from daily admissions to a 7-day rolling weekly sum to account for these potential day-of-week effects before calculating the forecastability score. The weekly comparison in Fig 6 downsamples from the 7-day rolling sum time series.**

**We have clarified this in the figure legend and the Methods section (lines 210-211): “Before calculating the forecastability metric, time series were converted from daily values to rolling weekly sums (7-day) to help account for potential day of week reporting effects.”**

4.3 Related to 1) and 2), I wonder whether showing some of the spectral analyses across time series, in addition to showing the summarized forecastability metrics (omega) may be interesting. In particular this may highlight the strength of annual vs semi-annual cycles for some pathogens and geography/pop size.
**Response: This is an interesting idea, but challenging to do concisely since our summary scatter plots represent combinations of two seasons x two diseases x 50+ locations. We have added a new Figure 3 (panels B &C) to show sample time series and power spectra for small and large population size targets that were used to calculate the forecastability metric. Hopefully this provides some additional intuition around the metric and provides some edge cases for what these time series might look like.**

4.4 The impact of population size is interesting – there seems to be a change in forecastability at very large population sizes. There could be two drivers: one is stochastic noise (the boring hypothesis), and the other one would be true differences in transmission by population size. It would be interesting to downsample the target data from some of the larger states, down to the size of the smallest states, and rerun the forecastability analysis. If the downsampled large states behave like the saemm states, then it is just about noise. If the downsampled large states still have higher forecastibility, then there is something specific to transmission (or reporting may be better in larger states?).
I think this merits a discussion because it is not intuitive that large populations should be more predictable. Large US states are made of quite large geographic areas, which could in theory experience asynchronous/uncorrelated epidemics that could be more difficult to predict.
**Response: Thank you for this comment. The downsampling is an interesting idea but would need to be done thoughtfully to preserve geographic proximity within a state. We do see similar patterns in forecastability for CA counties (Fig 2) compared with US states vs US (Fig 3 & 4), and we therefore tend to agree with you on the “boring hypothesis” since this pattern with forecastability seems to occur regardless of the specific scale of geographic aggregation. We have added text to this effect, while noting that there may be other underlying drivers (lines 448-471): “Our results also echoed prior work that found a relationship between population size of the forecast target and forecast performance (Fig S3 & S4) (Lopez et al. 2024; White et al. 2023). Forecastability scores increased with population size when looking across CA counties (Fig 2) and across US states (Fig 3, 4). This observed relationship between forecastability and population size potentially reflects general intuition, i.e., that larger populations should have signals closer to the mean field, with corresponding weaker high frequency components, resulting in a higher forecastability score…Together these two additional sub-analyses point to in improved signal-to-noise ratio arising from data aggregation—whether spatial (i.e., larger populations with larger reporting volume) or temporal—as a potential mechanism for a higher forecastability score, rather than some distinct transmission phenomenon occurring at specific scales.”**

**Please also see our response to Comment #2.4 above where we explore this question more by: (1) looking at the relationship between cumulative incidence, forecastability, and forecast performance; and (2) looking at forecastability for %ED visits which have an implicit denominator that reflects underlying volume.**

Additional comments:
4.5 The prediction performance analysis relies on 1- to 4-wk hub forecasts. Was there any difference in the relationship between forecastability and performance when the data is split by forecast horizon? Similarly did these relationships differ around the peak of the epidemic? (epidemic troughs may contribute to a lot of the signal shown here?)

**Response: We have added Figure 5 in the main text to address the question of dependence on forecast horizon. The relationship between forecastability and forecast performance was robust across different horizons (i.e., 1- to 4-weeks ahead).**

**Since we calculated the forecastability metric corresponding to the time series for each respiratory season (e.g., October- September), the forecast scores are averaged across all forecast targets during that interval. We agree that the question of whether these relationships differ at different periods of the epidemic is an interesting one. We have highlighted it as a future direction in the Discussion that could be done when implementing a rolling window approach (lines 536-540“It would also be interesting to see if the relationship between forecastability and forecast performance changes during periods of the epidemic, e.g., near the peak or during low levels of activity. For example, Mills et al. (2025) use a 12-week trailing window approach in combination with permutation entropy to look at localized forecast performance.”**

4.6 Conceptually, forecast performance reflects model accuracy both in terms of the reporting process and the disease dynamics, and it makes intuitive sense that having repeated patterns in either will improve forecast performance. Yet there are instances where having very little historic data (hence few repeat cycles) may help, as would be the case for an invasion wave of a new pathogen where immunity is low or non-existent. As contributors of the US Scenario hub, the authors will be familiar with the Omicron round, where Hub projections did particularly well on a 3-month horizon (predictions outperformed published short-term forecasts), presumably because the simple laws governing the SIR framework applied for an immune escape variant. With a good understanding of transmission and immunity mechanisms, one may not need to rely on predictable cycles. On the flipside, with a summarized measure like forecastability it can be difficult to pinpoint exactly what is predictable in the signal. Perhaps worth elaborating on an already good discussion?

**Response: This is a great conceptual point, and we have added some context to the Discussion, using your example from the Scenario Modeling Hub (lines 514-524): “The utility of this metric is based on the assumption that having repeated patterns in disease reporting or dynamics might be expected to improve forecast performance. However, there are also potential instances where disease spread of a novel pathogen or new variant in a completely naïve population—with little historical data or repeating cycles—could be well informed by using a mechanistic understanding of disease transmission alone. For example, during the Omicron round of the US COVID-19 Scenario Modeling Hub (Round 11), Hub projections provided accurate information about the timing and magnitude of the new variant (Howerton et al. 2023), presumably because the simple laws governing the SIR framework applied for an immune escape variant. Therefore, with a good understanding of transmission and immunity mechanisms, one may not need to rely on predictable cycles.”**

## References cited in response to reviewers:

Bandt, Christoph, and Bernd Pompe. 2002. “Permutation Entropy: A Natural Complexity Measure for Time Series.” *Physical Review Letters* 88(17): 174102. doi:10.1103/PhysRevLett.88.174102.

Borchering, Rebecca K., Matthew Biggerstaff, Lynnette Brammer, Alicia Budd, Shikha Garg, Alicia M. Fry, A. Danielle Iuliano, and Carrie Reed. 2024. “Responding to the Return of Influenza in the United States by Applying Centers for Disease Control and Prevention Surveillance, Analysis, and Modeling to Inform Understanding of Seasonal Influenza.” *JMIR Public Health and Surveillance* 10(1): e54340. doi:10.2196/54340.

Bosse, Nikos I., Sam Abbott, Anne Cori, Edwin van Leeuwen, Johannes Bracher, and Sebastian Funk. 2023. “Scoring Epidemiological Forecasts on Transformed Scales.” *PLOS Computational Biology* 19(8): e1011393. doi:10.1371/journal.pcbi.1011393.

Bosse, Nikos I., Hugo Gruson, Anne Cori, Edwin van Leeuwen, Sebastian Funk, and Sam Abbott. 2022. “Evaluating Forecasts with Scoringutils in R.” *arXiv*. doi:10.48550/ARXIV.2205.07090.

Cramer, Estee Y, Yuxin Huang, Yijin Wang, Evan L Ray, and Matthew Cornell. 2022. “The United States COVID-19 Forecast Hub Dataset.” *Scientfic Data* 9(1): 462.

Cramer, Estee Y., Evan L. Ray, Velma K. Lopez, Johannes Bracher, Andrea Brennen, Alvaro J. Castro Rivadeneira, Aaron Gerding, et al. 2022. “Evaluation of Individual and Ensemble Probabilistic Forecasts of COVID-19 Mortality in the United States.” *Proceedings of the National Academy of Sciences of the United States of America* 119(15): e2113561119. doi:10.1073/pnas.2113561119.

Frutos, Aaron M., Shunte Moon, Alison M. Binder, Andrea J. Cool, Kingsley Iyawe, Trevor Thompson, Margaret A. Dudeck, et al. 2025. “Evaluation of a Novel Data Source for National Influenza Surveillance: Influenza Hospitalization Data in the National Healthcare Safety Network, United States, September 2021–April 2024.” *American Journal of Public Health*: e1–5. doi:10.2105/AJPH.2025.308282.

Garland, Joshua, Ryan James, and Elizabeth Bradley. 2014. “Model-Free Quantification of Time-Series Predictability.” *Physical Review E* 90(5): 052910. doi:10.1103/PhysRevE.90.052910.

Goerg, Georg M. 2013. “Forecastable Component Analysis.” In *Proceedings of the 30 Th International Conference on Machine Learning*, Atlanta, Georgia.

Howerton, Emily, Lucie Contamin, Luke C. Mullany, Michelle Qin, Nicholas G. Reich, Samantha Bents, Rebecca K. Borchering, et al. 2023. “Evaluation of the US COVID-19 Scenario Modeling Hub for Informing Pandemic Response under Uncertainty.” *Nature Communications* 14(1): 7260. doi:10.1038/s41467-023-42680-x.

Kang, Yanfei, Rob J Hyndman, and Kate Smith-Miles. 2016. “Visualising Forecasting Algorithm Performance Using Time Series Instance Spaces.”

Lopez, Velma K., Estee Y. Cramer, Robert Pagano, John M. Drake, Eamon B. O’Dea, Madeline Adee, Turgay Ayer, et al. 2024. “Challenges of COVID-19 Case Forecasting in the US, 2020–2021.” *PLOS Computational Biology* 20(5): e1011200. doi:10.1371/journal.pcbi.1011200.

Mathis, Sarabeth M., Alexander E. Webber, Tomás M. León, Erin L. Murray, Monica Sun, Lauren A. White, Logan C. Brooks, et al. 2024. “Evaluation of FluSight Influenza Forecasting in the 2021-22 and 2022-23 Seasons with a New Target Laboratory-Confirmed Influenza Hospitalizations.” *Nature Communications* 15(1): 6289. doi:10.1038/s41467-024-50601-9.

Mills, Cathal, Nicholas J. Irons, Joseph L.-H. Tsui, Sarah Sparrow, Luiz M. Carvalho, Adam J. Kucharski, Oliver Ratmann, et al. 2025. “From Metric to Action: An Evaluation Framework to Translate Infectious Disease Forecasts into Policy Decisions.” : 2025.07.20.25331802. doi:10.1101/2025.07.20.25331802.

Papacharalampous, Georgia, Hristos Tyralis, Ilias G. Pechlivanidis, Salvatore Grimaldi, and Elena Volpi. 2022. “Massive Feature Extraction for Explaining and Foretelling Hydroclimatic Time Series Forecastability at the Global Scale.” *Geoscience Frontiers* 13(3): 101349. doi:10.1016/j.gsf.2022.101349.

Scarpino, Samuel V., and Giovanni Petri. 2019. “On the Predictability of Infectious Disease Outbreaks.” *Nature Communications* 10(1): 898. doi:10.1038/s41467-019-08616-0.

Scobie, Heather M., Mark Panaggio, Alison M. Binder, Molly E. Gallagher, William M. Duck, Philip Graff, and Benjamin J. Silk. 2023. “Correlations and Timeliness of COVID-19 Surveillance Data Sources and Indicators ― United States, October 1, 2020–March 22, 2023.” *MMWR. Morbidity and Mortality Weekly Report* 72(19): 529–35. doi:10.15585/mmwr.mm7219e2.

Shao, Zezhi, Fei Wang, Yongjun Xu, Wei Wei, Chengqing Yu, Zhao Zhang, Di Yao, et al. 2025. “Exploring Progress in Multivariate Time Series Forecasting: Comprehensive Benchmarking and Heterogeneity Analysis.” *IEEE Transactions on Knowledge and Data Engineering* 37(1): 291–305. doi:10.1109/TKDE.2024.3484454.

Sundararajan, D. 2025. “Aliasing and Leakage.” In *Fourier Analysis—A Signal Processing Approach*, ed. D. Sundararajan. Singapore: Springer Nature, 149–73. doi:10.1007/978-981-96-1078-5_5.

White, Lauren A., Ryan McCorvie, David Crow, Seema Jain, and Tomás M. León. 2023. “Assessing the Accuracy of California County Level COVID-19 Hospitalization Forecasts to Inform Public Policy Decision Making.” *BMC Public Health* 23(1): 782. doi:10.1186/s12889-023-15649-0.
